# KIR and their HLA Class I ligands: Two more pieces towards completing the puzzle of chronic rejection and graft loss in kidney transplantation

**DOI:** 10.1371/journal.pone.0180831

**Published:** 2017-07-07

**Authors:** Roberto Littera, Gianbenedetto Piredda, Davide Argiolas, Sara Lai, Elena Congeddu, Paola Ragatzu, Maurizio Melis, Elisabetta Carta, Maria Benigna Michittu, Donatella Valentini, Luisella Cappai, Rita Porcella, Francesco Alba, Maria Serra, Valentina Loi, Roberta Maddi, Sandro Orrù, Giorgio La Nasa, Giovanni Caocci, Roberto Cusano, Marcella Arras, Mauro Frongia, Antonello Pani, Carlo Carcassi

**Affiliations:** 1 Regional Transplant Center, R. Binaghi Hospital, ASSL Cagliari, ATS Sardegna, Italy; 2 Kidney Transplant Unit, Department of Renal Dieases, G. Brotzu Hospital, Cagliari, Italy; 3 Medical Genetics, R. Binaghi Hospital, ASSL Cagliari, ATS Sardegna, Italy; 4 Medical Genetics, Department of Medical Sciences and Public Health, University of Cagliari, Cagliari, Italy; 5 Bone Marrow Transplant Center, R. Binaghi Hospital, ASSL Cagliari, ATS Sardegna, Italy; 6 Hematology Unit, Department of Medical Sciences and Public Health, University of Cagliari, Cagliari, Italy; 7 Center for Advanced Studies, Research and Development (CRS4) Biomedical Sector, "Polaris" Technology Park, Pula, Cagliari, Italy; 8 Complex Structure of Urology, Kidney Transplantation and Robotic Surgery, G. Brotzu Hospital, Cagliari, Italy; 9 Complex Structure of Nephrology and Dialysis, Department of Renal Diseases, G. Brotzu Hospital, Cagliari, Italy; University of Toledo, UNITED STATES

## Abstract

**Background:**

Kidney transplantation is a life-saving treatment for patients with end-stage renal disease. However, despite progress in surgical techniques and patient management, immunological rejection continues to have a negative impact on graft function and overall survival. Incompatibility between donors and recipients for human leukocyte antigens (HLA) of the major histocompatibility complex (MHC) generates a series of complex cellular and humoral immune response mechanisms that are largely responsible for rejection and loss of graft function. Within this context, a growing amount of evidence shows that alloreactive natural killer (NK) cells play a critical role in the immune response mechanisms elicited by the allograft. Killer immunoglobulin-like receptors (KIRs) are prominent mediators of NK cell alloreactivity.

**Methods and findings:**

A cohort of 174 first cadaveric kidney allograft recipients and their donors were selected from a total cohort of 657 transplanted patients for retrospective immunogenetic analyses. Patients with HLA Class II mismatches were excluded. HLA Class I allele frequencies were compared among patients with chronic rejection, patients with stable graft function and a group of 2388 healthy controls. Activating and inhibitory KIR gene frequencies, KIR haplotypes, KIR-HLA ligand matches/mismatches and combinations of recipient KIRs and donor HLA Class I ligands were compared among patients with and without chronic rejection and a group of 221 healthy controls.

Patients transplanted from donors homozygous for HLA-C1 antigens had a significantly higher risk for chronic rejection than patients transplanted from donors homozygous or heterozygous for HLA-C2 antigens or with epitopes belonging to the HLA-Bw4 ligand group.

The Kaplan-Meier curves obtained by dividing the patients into 3 groups according to the presence or absence of one or both of the combinations of recipient KIRs and donor HLA ligands (rKIR2DL1/dHLA-C2 and rKIR3DL1/dHLA-Bw4) showed a significantly higher cumulative incidence of chronic rejection in the group of patients completely lacking these functional units. These patients showed a progressively stronger decline in modification of diet in renal disease-estimated glomerular filtration rate.

**Conclusions:**

KIR genotyping should be performed at the time of enrolment of patients on the waiting list for organ transplantation. In our study, a significantly higher risk of chronic rejection after kidney transplantation was observed when recipient (r) and donor (d) pairs completely lacked the two functional rKIR-dHLA ligand combinations rKIR2DL1/dHLA-C2 and rKIR3DL1/dHLA-Bw4. This immunogenetic profile corresponds to low levels of NK cell inhibition. Therefore, patients with this high risk profile could benefit from immunosuppressive therapy aimed at reducing NK-cell cytotoxicity.

## Introduction

Current advances in transplantation techniques and the improvement achieved in patient management through innovative approaches to immunosuppressive therapy and anti-infective prophylaxis, has resulted in a reduction of acute (AR) and chronic (CR) rejection episodes. Nonetheless, immunological rejection continues to cause loss of graft function and a decrease in overall survival rates [1, 2].

One of the main factors determining a successful outcome of kidney transplantation is the compatibility between the donor and recipient for human leukocyte antigen (HLA) genes of the major histocompatibility complex (MHC), particularly those of class II [3, 4]. The complex immunological mechanisms underlying acute and chronic rejection involve both humoral and cell-mediated immunity. Rejection of the allograft is largely dependent upon T cell response to HLA incompatibility. Alloreactive T cells recognize major and minor HLA histocompatibility antigens via their specific T cell receptors (TCR) and thereby trigger multiple and complex effector functions [2, 5, 6]. On the other hand, evidence continues to emerge that alloreactive natural killer (NK) cells also have a key role in immune response mechanisms elicited by the allograft. It has been shown that NK cells infiltrate kidney allografts [7–9] and that increased numbers of NK cells are found in the peripheral blood of patients acutely rejecting kidney graft [10]. Moreover, increased cytotoxicity of recipient NK cells against donor peripheral blood cells has been described in vitro [11].

Human NK cells express multiple receptors that interact with HLA class I molecules. These receptors pertain to either the immunoglobulin-like superfamily or the C-type-lectin-like receptor superfamily. Killer immunoglobulin-like receptors (KIRs) are key regulators of NK cell activity and predominantly recognize classical HLA class I molecules.

Historical nomenclature distinguishes two groups of KIR haplotypes. Group A haplotypes contain a fixed set of 7 KIR genes (the *KIR3DL3*, *KIR2DL1*, *KIR2DL3*, *KIR2DL4*, *KIR3DL1* and *KIR3DL2* inhibitory KIR genes and the *KIR2DS4* activating KIR gene) whereas Group B haplotypes embrace all other KIR haplotypes with different combinations of activating and inhibitory KIR genes and at least one of the specific KIR genes, *KIR2DS1*, *KIR2DS2*, *KIR2DS3*, *KIR2DS5*, *KIR3DS1*, *KIR2DL2* and *KIR2DL5* [12, 13]. It is noteworthy that up to 70% of Caucasian individuals who are homozygous for KIR haplotype A exclusively carry non-functional deletion variants of *KIR2DS4* [14] which means that they do not express activating KIRs on the NK cell surface.

KIR ligands are HLA-C and HLA-B molecules that have been divided into 3 different categories according to the amino acid sequence determining the KIR-binding epitope. HLA-C alleles have been assigned to HLA-C group 1 (C1) or HLA-C group 2 (C2), depending on whether there is an asparagine or lysine at position 80 of the alpha-1 domain of the alpha helix [15, 16]. Most HLA-B alleles can be distinguished by the presence of the serological epitopes Bw4 and Bw6 but only those bearing the HLA-Bw4 motif serve as ligands for KIRs.

The KIR2DL2 and KIR2DL3 inhibitory receptors and the KIR2DS2 activating receptor bind HLA-C1 KIR ligands while the KIR2DL1 inhibitory receptor and the KIR2DS1 activating receptor bind HLA-C2 KIR ligands. The KIR3DL1 inhibitory receptor binds the HLA-Bw4 KIR ligand. Several reports also suggest binding between the KIR3DS1 activating receptor and HLA-Bw4. However, it has recently been shown that the six amino acid differences in the external domains that distinguish KIR3DL1 from KIR3DS1 are sufficient to completely suppress interaction of KIR3DS1 with HLA molecules containing the HLA-Bw4 epitope. Indeed, there is no experimental evidence demonstrating that interaction between KIR3DS1 and HLA-Bw4 physically occurs [17]. Although it has been well established that this activating receptor is implicated in a large variety of human diseases, a ligand that would account for its biological effects remains unknown. Only very recently, Garcia-Beltran et al. demonstrated that KIR3DS1 binds to HLA-F, a result which they confirmed biochemically and functionally [18]. Other factors capable of influencing KIR-mediated NK cell licensing and inhibition are variations in KIR-ligand interactions and binding affinities. For example, it has been shown that some antigens of *KIR2DL2* and *KIR2DL3* are also capable of interacting with HLA-C2 ligands [19, 20].

Healthy cells expressing normal HLA Class I molecules are spared by inhibitory receptors on the NK cell surface. However, cells damaged by viral infection or neoplastic transformation may lose HLA Class I expression and be eliminated by NK cells.

Because HLA and KIR ligands are highly polymorphic, inhibitory KIRs expressed by NK cells of solid organ transplant recipients with donors mismatched for HLA KIR ligands, may not recognize HLA Class I molecules of donor cells and generate NK cell alloreactivity against the transplanted organ. A similar reaction in the opposite direction has been described in hematopoietic stem cell transplantation [21, 22]. Moreover, certain KIR genotypes together with their specific HLA Class I ligands could also influence kidney transplantation outcome by interfering with the efficacy of immunosuppressive therapies. Interference of KIR-ligand interactions with drug effectiveness has already been described in allogeneic transplantation of hematopoietic stem cells and onco-hematological disorders such as chronic myeloid leukemia [23–25].

KIRs are also expressed on a fraction of cytotoxic T cells such as CD8+ T cells, gamma delta T lymphocytes and especially CD4+CD28- cytotoxic T cells which have been implicated in the mechanisms leading to autoimmune vasculitis [26–28], suggesting a potential influence of KIRs on T-cell mediated anti-graft response.

Although the knowledge acquired so far has been impressive, many questions remain unanswered. In organ transplantation, interaction between HLA and KIRs expressed on donor and recipient cells and their role in NK cell-mediated alloreactivity requires further investigation.

In the present study, we investigated the clinical impact of these interactions on the development and course of chronic rejection with particular emphasis on recipient KIRs and donor HLA class I ligands.

## Materials and methods

From January 2005 to March 2014, 657 patients underwent kidney transplantation in the Organ Transplantation Center of the G. Brotzu Hospital in Cagliari, Italy. Documented negative results for standard complement-dependent cytotoxicity (CDC) T- and B-cell crossmatches were available for all patients. HLA Class II incompatibility is widely recognized as an important risk factor for antibody-mediated rejection, transplant glomerulopathy and allograft loss with HLA-DR mismatches having the greatest influence. For this reason, only recipient/donor pairs fully matched for the *HLA-DR* locus were included in our study. In order to further reduce potential bias, also patients transplanted from a living donor or receiving a second allograft were excluded as well as patients presenting de novo anti-HLA antibodies equal to or above the threshold of 1500 Luminex mean fluorescence intensity (MFI) at the time of transplantation.

Altogether, 483 patients were excluded from the study leaving a cohort of 174 first cadaveric kidney allograft recipients and their donors for retrospective immunogenetic analysis. All 174 patients were systematically investigated for the clinical and immunological parameters known to have a major influence on the transplantation outcome: number of HLA class I (0–2 HLA-A, 0–2 HLA-B) mismatches, panel reactive antibodies (PRA) >50%, graft cold ischemia time, induction therapy and post-graft immunotherapy.

One hundred and thirty-two of the 174 patients had stable graft function (SGF) on the date of the last follow-up examination. The remaining 42 patients presented clinical signs of progressive kidney dysfunction attributable to chronic rejection, as diagnosed by the evaluation of biopsies according to Banff 1997 criteria with the modifications of the 10th Banff Conference held in 2009 [29].

### Ethics statement

The study was performed in adherence to the Declaration of Helsinki. The study was approved by the Ethics Committee of the University of Cagliari: Protocol n. 2014/456 dated 23^rd^ of January 2014. Written informed consent was obtained from all patients and is kept on file in the pertinent Medical Record Office of the G. Brotzu Hospital in Cagliari, Italy.

### Laboratory measurements

The percentage of PRA levels was determined in pre-transplantation sera of all 174 patients using an enzyme-linked immunosorbent assay (ELISA) methodology detecting reactive immunoglobulin G (IgG) against a panel of 81 HLA class I antigens and 47 HLA class II antigens (Lambda Antigen Tray Class I & II; One Lambda Inc., Canoga Park, CA, USA). Patients with PRA levels exceeding 50% were considered to be presensitized.

The glomerular filtration rate (GFR) was calculated by the abbreviated 4-variable Modification of Diet in Renal Disease (MDRD) study equation [GFR = 175 × standardized scr–1.154 × age–0.203 × 1.212 (if black) × 0.742 (if female), where scr refers to serum creatinine measured in mg/dL]. This version of the formula includes the variables of age, sex, serum creatinine and ethnicity, and is currently the preferred method in nephrology clinical trials [30].

### HLA and KIR genotyping

High resolution typing of the *HLA-A*, -*B*, -*C* and *DR* loci was performed in patients and their donors using a polymerase chain reaction sequence-specific primer (PCR-SSP) method according to the manufacturer’s instructions (Allele-specific PCR-SSP kits: Olerup SSP AB, Stockholm, Sweden).

HLA-C alleles were assigned to the C1 or C2 ligand category on the basis of the dimorphism (asparagine or lysine) at position 80 in the alpha helix.

HLA-B alleles were classified as either Bw4 or Bw6 according to the amino acid positions spanning positions 77–83. HLA-A23, -A24, and -A32 belong to the HLA-Bw4 group of serological epitopes.

Genomic DNA of patients and their donors was typed for the 14 KIR genes *KIR2DL1*, *KIR2DL2*, *KIR2DL3*, *KIR2DL4*, *KIR2DL5*, *KIR3DL1*, *KIR3DL2*, *KIR3DL3*, *KIR2DS1*, *KIR2DS2*, *KIR2DS3*, *KIR2DS4*, *KIR2DS5* and *KIR3DS1* using PCR with locus-specific primers as previously described [15, 31]. A group of 221 healthy controls underwent the same immunogenetic evaluation as the patients and their donors.

KIR2DS4 alleles bearing a 22-base pair (bp) deletion in exon 5 (KIR2DS4 non-functional deletion variants) were distinguished from functional KIR2DS4 alleles (KIR2DS4 full-length) using the method and set of primers described by Yawata et al. [32].

### Presence or absence of KIR-ligand mismatches, activating and inhibitory KIRs, KIR genotypes, KIR haplotype assignment and KIR-ligand combinations

KIR-ligand mismatches were defined according to Ruggeri et al. [33], considering that a mismatch requires the presence of the corresponding KIR to be detected. For example, a KIR2DL1-ligand mismatch requires the presence of the *KIR2DL1* gene and an HLA-C group 2 allele (C2+) in the recipient, combined with the absence of HLA-C group 2 alleles in the donor (C2-). One mismatch was sufficient to classify the kidney transplant as KIR mismatched, since also in this case NK-cell mediated alloreactivity against the allograft may occur [34–36].

Association of the number of activating or inhibitory KIRs with a potential effect on allograft outcome was investigated, taking into account that activating KIRs are encoded by *KIR2DS1*, *KIR2DS2*, *KIR2DS3*, *KIR2DS4*, *KIR2DS5* and *KIR3DS1* whereas inhibitory KIRs are encoded by *KIR2DL1*, *KIR2DL2*, *KIR2DL3*, *KIR2DL5*, *KIR3DL1*, *KIR3DL2* and *KIR3DL3*. It has been reported that *KIR2DL4* can exert both activating and inhibitory functions [37].

KIR B haplotypes typically possess one or more of the following genes: *KIR2DL5*, *KIR2DS1*, *KIR2DS2*, *KIR2DS3*, *KIR2DS5* and *KIR3DS1*. Because these genes are not present on KIR A haplotypes, patients homozygous for KIR A haplotypes (KIR genotype AA) could be distinguished from patients heterozygous or homozygous for KIR B haplotypes (KIR genotypes AB or BB, referred together as KIR genotype Bx) [13, 15].

Particular attention was dedicated to KIR-ligand combinations because of their essential role in the process of licensing that leads to the maturation of functional and potentially alloreactive NK cell clones [38, 39].

### Statistical analysis

Summary statistics were calculated on the clinical and biochemical data of kidney transplant patients diagnosed with or without chronic rejection: interquartile ranges (IQR), medians, mean differences and standard deviations (SD) were calculated on all continuous variables; percentages and odds ratios (OR) were calculated on categorical data. P values and 95% confidence intervals (95% CI) were obtained using Student’s t test or Fisher’s two-tailed exact test, as appropriate.

HLA Class I allele frequencies were compared between 174 donors and a large group of 2388 Sardinian healthy controls, as well as between donors of patients with chronic rejection and donors of patients with stable graft function. Statistical significance was calculated using Fisher’s two-tailed exact test. Only P values below 0.05 were considered to be statistically relevant.

KIR gene frequencies, KIR haplotypes, HLA-KIR ligands and combinations of recipient KIRs and donor HLA Class I ligands were compared among the different groups of patients and the group of 221 healthy controls. The incidence of chronic rejection was also investigated in relationship to single KIR-ligand mismatches/matches. The odds ratios, P values and 95% CI were calculated using Fisher’s two-tailed exact test. The threshold of significance was 0.05.

Kaplan-Meier curves were used to illustrate the cumulative incidence of chronic rejection after the date of transplantation up to the date of clinical, histopathological, and immuno-histochemical detection or the date of the last follow-up or death with a functioning graft. Recipient and donor pairs were stratified in three groups according to whether they had both, one or none of the following combinations of recipient (r) KIRs and donor (d) HLA ligands: rKIR2DL1/dHLA-C2 and rKIR3DL1/dHLA-Bw4. This made it possible to distinguish between patients with high, partial or low levels of NK cell inhibition, respectively. The log-rank test was used for comparisons of the different rKIR/dHLA-ligand combinations.

The rKIR2DL2/L3-dHLA-C1 combinations, well known to be an important axis of NK cell-target interactions in transplantation biology, were also investigated for association with chronic rejection in our study.

The mean GFR in the patients grouped according to high, partial or low NK cell inhibition was measured at 1, 6, 12, 24, 32, 48, 60 and 72 months from the date of transplantation. The group of patients with low NK cell inhibition was compared to the other two groups (high and partial NK cell inhibition) by calculating the area under the curve (AUC). Student’s t test was used to establish statistical significance [40]. The AUC was calculated according to the trapezium formula $\frac{1}{2}\Sigma_{i}\left(t_{i+1}-t_{i}\right)\left(y_{i}+y_{i+1}\right)$, where *t*_*i*_ is the time and *y*_*i*_ the filtration value. The summation was extended to include all times at which GFR was measured.

Creatinine serum values were assessed at 1, 6, 12, 24, 32, 48, 60 and 72 months after kidney transplantation. Comparisons between patients with low NK cell inhibition and patients with high or partial NK cell inhibition were performed at each measurement time using Student’s t test.

Statistical analysis was performed using R version 3.3.2 (The R Foundation for Statistical Computing, Vienna Austria).

## Results

One hundred and seventy-four patients (26.5%) were selected from a total cohort of 657 subjects who underwent kidney transplantation from January 2005 to March 2014. One hundred and thirty-two (75.9%) of the patients included in our study never presented clinical or laboratory signs of organ damage. The remaining 42 patients showed a progressive decline in graft function attributable to chronic immunological rejection, confirmed in all cases by renal biopsies. The histological findings in all these patients were characterized by tubular atrophy and interstitial fibrosis. Most patients (79%) also presented interstitial lymphomonocyte infiltration. Only a few patients presented arterial intima infiltrates. Fifteen of the 42 patients showed the presence of C4d on biopsies. None of the patients had a clinical history of calcineurin inhibitor toxicity, hypertensive damage, BK virus or bacterial infections. All patients were compliant with post-transplantation immunosuppressive therapy.

The age, sex, clinical and immunological characteristics of the recipient/donor pairs are given in Table 1.

**Table 1:** Clinical and immunological characteristics of kidney transplant patients.

	Patients	SGF	Chronic Rejection	P value; OR^(a)^ or x_1_-x_2_^(b)^ (95% CI)
**Kidney transplant patients**	174	132	42	
**Patient age, years (median, IQR)**	50 (43–62)	51 (43–61)	50 (40–64)	0.971; 0.1^(b)^ (-4.6 to 4.8)
**Donor age, years (median, IQR)**	47 (33–59)	48 (28–59)	47 (39–63)	0.143; -4.7^(b)^ (-11.1 to 1.6)
**Male patients, n (%)**	116 (66.6)	83 (62.9)	31 (73.8)	0.263; 0.6^(a)^ (0.3 to 1.4)
**Male donors, n (%)**	99 (56.8)	80 (60.6)	20 (47.6)	0.154; 1.7^(a)^ (0.8 to 3.6)
**Cytomegalovirus serostatus, donor/recipient, n (%)**				
negative/negative	5 (2.9)	3 (2.3)	2 (4.8)	0.595; 0.5^(a)^ (0.1 to 5.8)
negative/positive	26 (15.0)	19 (14.4)	7 (16.7)	0.804; 0.8^(a)^ (0.3 to 2.6)
positive/negative	10 (5.7)	7 (5.3)	3 (7.1)	0.706; 0.7^(a)^ (0.2 to 4.6)
positive/positive	133 (76.4)	103 (78.0)	30 (71.4)	0.407; 1.4^(a)^ (0.6 to 3.3)
**Patient/donor HLA compatibility** (mean ± SD)				
Class I (HLA-A, -B) mismatch (0–4)	2.9 ± 2.4	3.0 ± 2.5	2.4 ± 2.2	0.166; 0.6^(b)^ (-0.3 to 1.5)
Class II (HLA-DR) mismatch (0–2)	0	0	0	
**PRA, n (%)**				
Positivity for anti-HLA Class I antibodies >50%	9 (5.2)	4 (3.0)	5 (11.9)	0.038; 0.2^(a)^ (0.1 to 1.2)
Positivity for anti-HLA Class II antibodies >50%	10 (5.7)	5 (3.8)	5 (11.9)	0.063; 0.3^(a)^ (0.1 to 1.4)
**Antithymocyte globulin (ATG) induction therapy, n (%)**	16 (9.2)	10 (7.6)	6 (14.3)	0.221; 0.5^(a)^ (0.2 to 1.8)
Therapy, n (%)				
CsA/Tac-Evl/Srl-S	121 (69.5)	95 (72.0)	26 (61.9)	0.250; 1.6^(a)^ (0.7 to 3.5)
CsA/Tac-MMF-S	24 (13.8)	16 (12.1)	8 (19.1)	0.304; 0.6^(a)^ (0.2 to 1.7)
Evl/Srl-MMF-S	22 (12.6)	16 (12.1)	6 (14.3)	0.790; 0.8^(a)^ (0.3 to 2.8)
Other	7 (4.1)	5 (3.8)	2 (4.7)	0.676; 0.8^(a)^ (0.1 to 8.6)
**Cold ischemia time, hours (mean ± SD)**	14.1 ± 5.4	13.7 ± 5.2	15.2 ± 6.2	0.122; -1.5^(b)^ (-3.4 to 0.4)
**Known causes for renal insufficiency, n (%)**	132 (75.9)	101 (76.5)	31 (73.8)	0.836; 1.2^(a)^ (0.5 to 2.7)
Hypertension or renal vascular disease, n (%)	65 (37.4)	52 (39.4)	13 (31.0)	0.364; 1.5^(a)^ (0.7 to 3.3)
Glomerulonephritis, n (%)	49 (28.2)	34 (25.7)	15 (35.7)	0.239; 0.6^(a)^ (0.3 to 1.4)
Adult polycystic kidney disease, n (%)	37 (21.3)	29 (22.0)	8 (19.0)	0.830; 1.2^(a)^ (0.5 to 3.3)
Other, n (%)	23 (13.1)	17 (12.9)	6 (14.3)	0.797; 0.9^(a)^ (0.3 to 3.0)
**Transplantation outcome**				
Delayed graft function, days (mean ± SD)	1.5 ± 3.6	1.5 ± 3.4	1.4 ± 4.1	0.875; 0.1^(b)^ (-1.2 to 1.4)
GFR at 1 year, ml/min/1.73m2, MDRD study equation (mean ± SD)	56.9 ± 16.6	61.4 ± 16.6	42.6 ± 16.4	1.3·10^−9^; 18.8^(b)^ (13.0 to 24.6)
Serum creatinine at 1 year, mg/dl (mean ± SD)	1.4 ± 0.4	1.3 ± 0.3	1.8 ± 0.6	2.0·10^−11^; -0.5^(b)^ (-0.6 to -0.4)
One-year graft survival, n (%)	174 (100)	132 (100)	42 (100)	1
GFR at 3 years, ml/min/1.73m2, MDRD study equation (mean ± SD)	59.3 ± 19.9	64.9 ± 20.6	41.6 ± 17.5	4.7·10^−10^; 23.3^(b)^ (16.3 to 30.3)
Serum creatinine at 3 years, mg/dl (mean ± SD)	1.5 ± 0.5	1.3 ± 0.4	2.0 ± 1.0	5.4·10^−10^; -0.7^(b)^ (-0.9 to -0.5)
Three-year graft survival, n (%)	157 (90.2)	127 (96.2)	30 (71.4)	2.4·10^−5^; 10.0^(a)^ (3.0 to 39.0)

^(a)^ Odds ratios (for categorical variables): patients with stable graft function versus patients with chronic rejection.

^(b)^ Mean differences (for continuous variables): x_1_ (patients with stable graft function)–x_2_ (patients with chronic rejection).

IQR = interquartile range, CI = Confidence Interval, OR = Odds ratio, SD = standard deviation, Srl = sirolimus, S = steroids,

CsA = cyclosporin A, MMF = mycophenolate mofetil, Evl = everolimus, Tac = tacrolimus, PRA = panel-reactive antibody,

GFR = glomerular filtration rate, MDRD = Modification of diet in renal disease.

No significant differences were found between the two groups of patients with or without rejection for age and gender of the donors and recipients. The patients had a median age of 50 years (IQR 43–62) and were prevalently males (66.6%). The donors had a median age of 47 years (IQR 33–59). Cytomegalovirus (CMV) serostatus was similar in the two groups. Also the causes determining renal insufficiency were similar. Moreover, there was considerable overlap between the treatment administered before and after transplantation (Table 1).

Induction therapy was performed with antithymocyte globulin (ATG) in 9.2% of the cases with a higher percentage in the group with chronic rejection versus those with stable graft function (6/42, 14.3% vs 10/132, 7.6%). One hundred and twenty-one patients (69.5%) received post-transplantation immunosuppressive therapy according to a protocol based on sirolimus (rapamycin) or everolimus and steroids with or without cyclosporin or tacrolimus. Mycophenolate mofetil was administered to 46 patients (26.4%) either in combination with steroids or other immunosuppressive agents (cyclosporin or tacrolimus/everolimus or sirolimus). Overall, differences in immunosuppressive treatment schemes did not seem to influence the development of chronic rejection.

A higher percentage of hyperimmunized patients (PRA >50% against HLA Class I and II antigens) was found after transplantation in the group of patients with chronic rejection compared to the group of patients with stable graft function. This difference was only significant for HLA Class I antibodies [11.9% (5/42) vs 3.0% (4/132), OR = 0.2, 95% CI 0.1–1.2, P = 0.038; Table 1].

The number of HLA Class I (HLA-A, -B) mismatches (0–4) was not significantly different between the two groups.

The 1-year graft survival rate was 100% in both groups of patients, whereas the 3-year graft survival rate was lower in patients with chronic rejection compared to those with stable graft function (OR = 10.0, 95% CI 3.0–39.0, P = 2.4 x 10^−5^). Statistically significant differences were also observed for serum creatinine levels and GFR between patients with chronic rejection and those with stable graft function both at one and three years after transplantation (Table 1).

Analysis of Class I HLA loci revealed considerable overlap between the allele frequencies of the 174 recipient and donor pairs and a group of 2388 healthy controls from the Sardinian Voluntary Bone Marrow Donor Registry (S1 Table). Interestingly, certain HLA class I antigens of the kidney grafts of the 42 patients with chronic rejection had a higher frequency when compared to those of the kidney grafts of 132 patients with stable graft function, particularly HLA-A*24 and HLA-B*14 (HLA-A*24: 23.8% vs 5.3%, P = 5.0 x 10^−6^ and HLA-B*14: 16.7% vs 5.3%, P = 0.002; Table 2).

**Table 2:** HLA Class I allele frequencies in cadaveric kidney donors and healthy controls.

	HLA (2388 controls; 4776 alleles) n (%)	HLA (174 donors; 348 alleles) n (%)	Controls vs Donors P value	Donor HLA		
				(132 donors of patients with SGF; 264 alleles) n (%)	(42 donors of patients with CR; 84 alleles) n (%)	SGF vs CR P value
**HLA-A allele frequencies**						
-A*01	363 (7.6)	24 (6.9)	0.752	16 (6.1)	8 (9.5)	0.322
-A*02	1256 (26.3)	104 (29.9)	0.148	86 (32.6)	18 (21.4)	0.056
-A*03	282 (5.9)	20 (5.7)	1	16 (6.1)	4 (4.8)	0.792
-A*11	397 (8.3)	28 (8.0)	0.920	24 (9.1)	4 (4.8)	0.254
-A*23	82 (1.7)	0 (0)	0.006	-	-	-
-A*24	444 (9.3)	34 (9.8)	0.774	14 (5.3)	20 (23.8)	**5.0·10**^**−6**^
-A*26	134 (2.8)	16 (4.6)	0.068	16 (6.1)	0 (0)	0.016
-A*29	93 (1.9)	2 (0.6)	0.065	0 (0)	2 (2.4)	0.058
-A*30	851 (17.8)	52 (14.9)	0.190	40 (15.2)	12 (14.3)	1
-A*31	66 (1.4)	4 (1.1)	1	2 (0.8)	2 (2.4)	0.247
-A*32	419 (8.8)	34 (9.8)	0.495	30 (11.4)	4 (4.8)	0.092
-A*33	193 (4.0)	20 (5.7)	0.126	12 (4.5)	8 (9.5)	0.106
-A*68	96 (2.0)	4 (1.1)	0.320	4 (1.5)	0 (0)	0.576
-A*69	51 (1.1)	0 (0)	0.048	-	-	-
-A-	49 (1.0)	6 (1.7)	0.271	4 (1.5)	2 (2.4)	0.634
**HLA-C allele frequencies**						
-Cw*01	161 (3.4)	16 (4.6)	0.223	8 (3.0)	8 (9.5)	**0.030**
-Cw*02	299 (6.3)	20 (5.7)	0.818	16 (6.1)	4 (4.8)	0.792
-Cw*03	266 (5.6)	20 (5.7)	0.904	16 (6.1)	4 (4.8)	0.792
-Cw*04	633 (13.3)	56 (16.1)	0.143	42 (15.9)	14 (16.7)	0.866
-Cw*05	838 (17.5)	70 (20.1)	0.244	58 (22.0)	12 (14.3)	0.159
-Cw*06	320 (6.7)	28 (8.0)	0.321	22 (8.3)	6 (7.1)	0.822
-Cw*07	1095 (22.9)	80 (23.0)	1	58 (22.0)	22 (26.2)	0.457
-Cw*08	360 (7.5)	24 (6.9)	0.752	14 (5.3)	10 (11.9)	**0.048**
-Cw*12	364 (7.6)	24 (6.9)	0.676	22 (8.3)	2 (2.4)	0.081
-Cw*14	66 (1.4)	0 (0)	0.022	-	-	-
-Cw*15	221 (4.6)	10 (2.9)	0.141	8 (3.0)	2 (2.4)	1
-Cw*16	102 (2.1)	0 (0)	0.001	-	-	-
-Cw*17	51 (1.1)	0 (0)	0.048	-	-	-
**HLA-B allele frequencies**						
-B*07	132 (2.8)	4 (1.1)	0.082	2 (0.8)	2 (2.4)	0.247
-B*08	92 (1.9)	6 (1.7)	1	2 (0.8)	4 (4.8)	**0.032**
-B*13	109 (2.3)	8 (2.3)	1	8 (3.0)	0 (0)	0.207
-B*14	369 (7.7)	28 (8.0)	0.835	14 (5.3)	14 (16.7)	**0.002**
-B*15	88 (1.8)	0 (0)	0.004	-	-	-
-B*18	1066 (22.3)	82 (23.6)	0.594	64 (24.2)	18 (21.4)	0.660
-B*27	113 (2.4)	6 (1.7)	0.580	4 (1.5)	2 (2.4)	0.634
-B*35	661 (13.8)	60 (17.2)	0.079	42 (15.9)	18 (21.4)	0.249
-B*37	65 (1.4)	8 (2.3)	0.156	8 (3.0)	0 (0)	0.207
-B*38	80 (1.7)	12 (3.4)	0.032	10 (3.8)	2 (2.4)	0.738
-B*39	103 (2.2)	14 (4.0)	0.038	12 (4.5)	2 (2.4)	0.532
-B*40	77 (1.6)	0 (0)	0.010	-	-	-
-B*41	53 (1.1)	2 (0.6)	0.585	2 (0.8)	0 (0)	1
-B*44	221 (4.6)	12 (3.4)	0.353	12 (4.5)	0 (0)	0.078
-B*45	54 (1.1)	2 (0.6)	0.588	2 (0.8)	0 (0)	1
-B*49	217 (4.5)	14 (4.0)	0.789	12 (4.5)	2 (2.4)	0.532
-B*50	59 (1.2)	8 (2.3)	0.134	8 (3.0)	0 (0)	0.207
-B*51	343 (7.2)	18 (5.2)	0.192	14 (5.3)	4 (4.8)	1
-B*52	83 (1.7)	6 (1.7)	1	6 (2.3)	0 (0)	0.342
-B*55	178 (3.7)	10 (2.9)	0.553	6 (2.3)	4 (4.8)	0.262
-B*57	43 (0.9)	0 (0)	0.116	-	-	-
-B*58	436 (9.1)	28 (7.8)	0.562	22 (8.3)	6 (7.1)	0.822
-B-	134 (2.8)	20 (5.6)	0.005	14 (5.3)	6 (7.1)	0.590

SGF = stable graft function, CR = chronic rejection

Table 3 shows the frequencies of activating and inhibitory KIR genes and KIR haplotypes in 174 kidney transplant patients and 221 healthy controls. No significant differences were observed between the two groups. In comparisons between patients with chronic rejection and patients with stable graft function, a significant difference was only observed for the *KIR3DL1* inhibitory KIR gene which was present with a lower frequency in the group of patients with chronic rejection (85.7% vs 95.5%, OR 0.29, 95% CI 0.09–0.94, P = 0.041). We also analyzed the frequencies of the deletion (DV) and full-length (FL) variants of the *KIR2DS4* gene in the two groups of patients and healthy controls but did not find any significant differences [*KIR2DS4* DV/DV: SGF 79 (62.7%) vs CR 31 (77.5%), P = 0.141; *KIR2DS4* DV/FL: SGF 27 (21.4%) vs CR 5 (12.5%), P = 0.259; *KIR2DS4* FL/FL: SGF 20 (15.9%) vs CR 4 (10.0%), P = 0.448]. Therefore, we excluded *KIR2DS4* gene variants as having a potential role in the development of chronic rejection. Presence or absence of the well-characterized KIR A and B haplotypes had no influence on the development of chronic rejection. Higher numbers of activating KIR genes did not correlate with worse outcomes of kidney transplantation (Table 3).

**Table 3:** KIR gene frequencies and KIR haplotypes in patients and 221 healthy controls.

	221 controls n (%)	174 patients n (%)	P value	132 SGF n (%)	42 CR n (%)	P value	OR (95% CI)
**Patient inhibitory KIR genes**							
**2DL1**	215 (97.3)	168 (96.6)	0.771	126 (95.5)	42 (100)	0.338	-
**2DL2**	126 (57.0)	98 (56.3)	0.919	78 (59.1)	20 (47.6)	0.214	0.63 (0.29–1.34)
**2DL3**	188 (85.1)	154 (88.5)	0.373	120 (90.9)	34 (81.0)	0.096	0.43 (0.51–1.31)
**2DL4**	221 (100)	174 (100)	1	132 (100)	42 (100)	1	-
**2DL5**	116 (52.5)	106 (60.9)	0.103	82 (62.1)	24 (57.1)	0.590	0.81 (0.38–1.76)
**3DL1**	206 (93.2)	162 (93.1)	1	126 (95.5)	36 (85.7)	**0.041**	**0.29 (0.09–0.94)**
**3DL2**	219 (99.1)	174 (100)	0.506	132 (100)	42 (100)	1	-
**3DL3**	221 (100)	174 (100)	1	132 (100)	42 (100)	1	-
**Patient activating KIR genes**							
**2DS1**	97 (43.9)	94 (54.0)	0.054	73 (55.3)	21 (50.0)	0.723	0.81 (0.38–1.76)
**2DS2**	125 (56.6)	90 (51.7)	0.361	70 (53.0)	20 (47.6)	0.597	0.81 (0.38–1.71)
**2DS3**	77 (34.8)	58 (33.3)	0.831	46 (34.8)	12 (28.6)	0.573	0.75 (0.32–1.68)
**2DS4**	202 (91.4)	166 (95.4)	0.159	126 (95.5)	40 (95.2)	1	0.95 (0.16–10.02)
2DS4 FL/FL	23 (11.4)	24 (14.4)	0.348	20 (15.9)	4 (10.0)	0.448	0.59 (0.14–1.92)
2DS4 FL/DV	50 (24.8)	32 (19.3)	0.320	27 (21.4)	5 (12.5)	0.259	0.53 (0.15–1.53)
2DS4 DV/DV	129 (63.8)	110 (66.3)	0.352	79 (62.7)	31 (77.5)	0.141	1.88 (0.83–4.53)
**2DS5**	75 (33.9)	66 (37.9)	0.459	46 (34.8)	20 (47.6)	0.148	1.69 (0.79–3.64)
**3DS1**	88 (39.8)	64 (36.8)	0.603	46 (34.8)	18 (42.9)	0.364	1.40 (0.64–3.01)
**Haplotypes**							
**AA**	66 (29.9)	48 (27.6)	0.656	36 (27.3)	12 (28.6)	0.846	1.07 (0.45–2.43)
**Bx**	155 (70.1)	126 (72.4)	0.656	96 (72.7)	30 (71.4)	0.846	0.94 (0.41–2.24)

FL = full length functional KIR2DS4 alleles, DV = non-functional deletion variants of KIR2DS4 alleles

Each KIR-ligand mismatch was analyzed separately or in combination with 0, 1 or more than 1 mismatch. Mismatch of the combinations rKIR2DL2/L3+/dHLAC1- was present in 18.8% of the patients with chronic rejection and 7% of the patients with stable graft function. However, this difference did not reach statistical significance [18.8% (6/32) vs 7% (7/100), OR 3.03, 95% CI 0.8–11.6, P = 0.082]. No correlation was found for single or multiple KIR-ligand mismatches [0 vs 1 or more KIR-ligand mismatches 20.2% (24/119) vs 32.7% (18/55), OR 1.92, 95% CI 0.9–4.2, P = 0.087]. However, this result may be biased by the small number of cases available for analysis (Table 4).

**Table 4:** KIR-HLA ligand mismatches/matches in the 174 recipient/donor pairs.

Recipient KIR/HLA Ligand	n (%)	Donor HLA	Number of transplants	Number of rejections	P value OR (95% CI)
KIR2DL1+/C2+	144 (82.7)	Mismatch C2– Match C2+	17 127	6 25	0.204 2.21 (0.61–7.31)
KIR2DL2/L3+/C1+	132 (75.9)	Mismatch C1– Match C1+	13 119	6 26	0.082 3.03 (0.77–11.60)
KIR3DL1+/Bw4+	131 (75.3)	Mismatch Bw4– Match Bw4+	14 117	0 14	0.361 0 (0–2.54)
KIR3DL2+/A3+ or A11+	60 (34.5)	Mismatch A3– and A11– Match A3+ or A11+	47 13	16 1	0.086 6.05 (0.76–280.65)
Recipient/donor	174 (100)	1 or more Mismatches 0 Mismatches	55 119	18 24	0.087 1.92 (0.87–4.18)

OR = odds ratio, CI = confidence interval,

+ = present,

– = absent

One of the most relevant findings emerging from our study was the impact of differences in frequencies of the HLA-C1, -C2, and -Bw4 donor KIR ligands between patients with chronic rejection and those with stable graft function (Table 5).

**Table 5:** Donor HLA KIR ligands and KIR-HLA ligand combinations in recipient/donor pairs.

	221 Controls n (%)	174 Patients n (%)	P value	132 Patients with SGF n (%)	42 Patients with CR n (%)	P value	OR (95% CI)
***Donor HLA KIR ligands***							
**C1/C1**	53 (24.0)	32 (18.4)	0.218	18 (13.6)	14 (33.3)	**0.006**	**3.14 (1.28–7.64)**
**C2/C2**	60 (27.1)	42 (24.1)	0.563	32 (24.2)	10 (23.8)	1	0.98 (0.38–2.32)
**C1/C2**	108 (48.9)	100 (57.5)	0.104	82 (62.1)	18 (42.9)	**0.032**	**0.46 (0.21–0.98)**
**HLA-Bw4**	175 (79.2)	124 (71.3)	0.077	100 (75.8)	24 (57.1)	**0.030**	**0.43 (0.19–0.95)**
**HLA-Bw6**	206 (93.2)	156 (89.7)	0.272	116 (87.9)	40 (95.2)	0.248	2.75 (0.60–25.67)
***Recipient activating KIR genes /donor HLA ligands***							
**2DS1/HLA-C2**	85 (38.5)	84 (48.3)	0.052	68 (51.5)	16 (38.1)	0.157	0.58 (0.26–1.24)
**2DS2/HLA-C1**	75 (33.9)	68 (39.1)	0.294	52 (39.4)	16 (38.1)	1	0.95 (0.43–2.04)
**3DS1/HLA-Bw4**	66 (29.9)	48 (27.6)	0.656	34 (25.8)	14 (33.3)	0.428	1.44 (0.62–3.22)
***Recipient inhibitory KIR genes /donor HLA ligands***							
**2DL1/HLA-C2**	163 (73.8)	138 (79.3)	0.234	110 (83.3)	28 (66.7)	**0.028**	**0.40 (0.17–0.96)**
**2DL2/HLA-C1**	78 (35.3)	76 (43.7)	0.097	60 (45.5)	16 (38.1)	0.476	0.74 (0.34–1.59)
**2DL3/HLA-C1**	135 (61.1)	114 (65.6)	0.401	90 (68.2)	24 (57.1)	0.198	0.62 (0.29–1.36)
**3DL1/HLA-Bw4**	159 (71.9)	116 (66.7)	0.272	96 (72.7)	20 (47.6)	**0.004**	**0.34 (0.16–0.74)**

SGF = stable graft function, CR = chronic rejection, OR = odds ratio, CI = confidence interval

Indeed, donor homozygosity for the HLA-C1 ligand group (C1/C1) was significantly higher in patients with chronic rejection (33.3% vs 13.6%, OR 3.14, 95% CI 1.28–7.64, P = 0.006). Conversely, the frequencies of donors heterozygous for the HLA-C1 and -C2 ligand groups (C1/C2) or with epitopes pertaining to the HLA-Bw4 ligand group were significantly reduced in these patients (42.9% vs 62.1%, OR 0.46, 95% CI 0.21–0.98, P = 0.032 and 57.1% vs 75.8%, OR 0.43, 95% CI 0.19–0.95, P = 0.030, respectively; Table 5).

An even more interesting finding was the significantly reduced presence of the rKIR2DL1/dHLA-C2 and rKIR3DL1/dHLA-Bw4 KIR-ligand combinations in patients with chronic rejection compared to those with stable graft function (66.7% vs 83.3%, OR 0.40, 95% CI 0.17–0.96, P = 0.028 and 47.6% vs 72.7%, OR 0.34, 95% CI 0.16–0.74, P = 0.004, respectively; Table 5).

**Fig 1:**
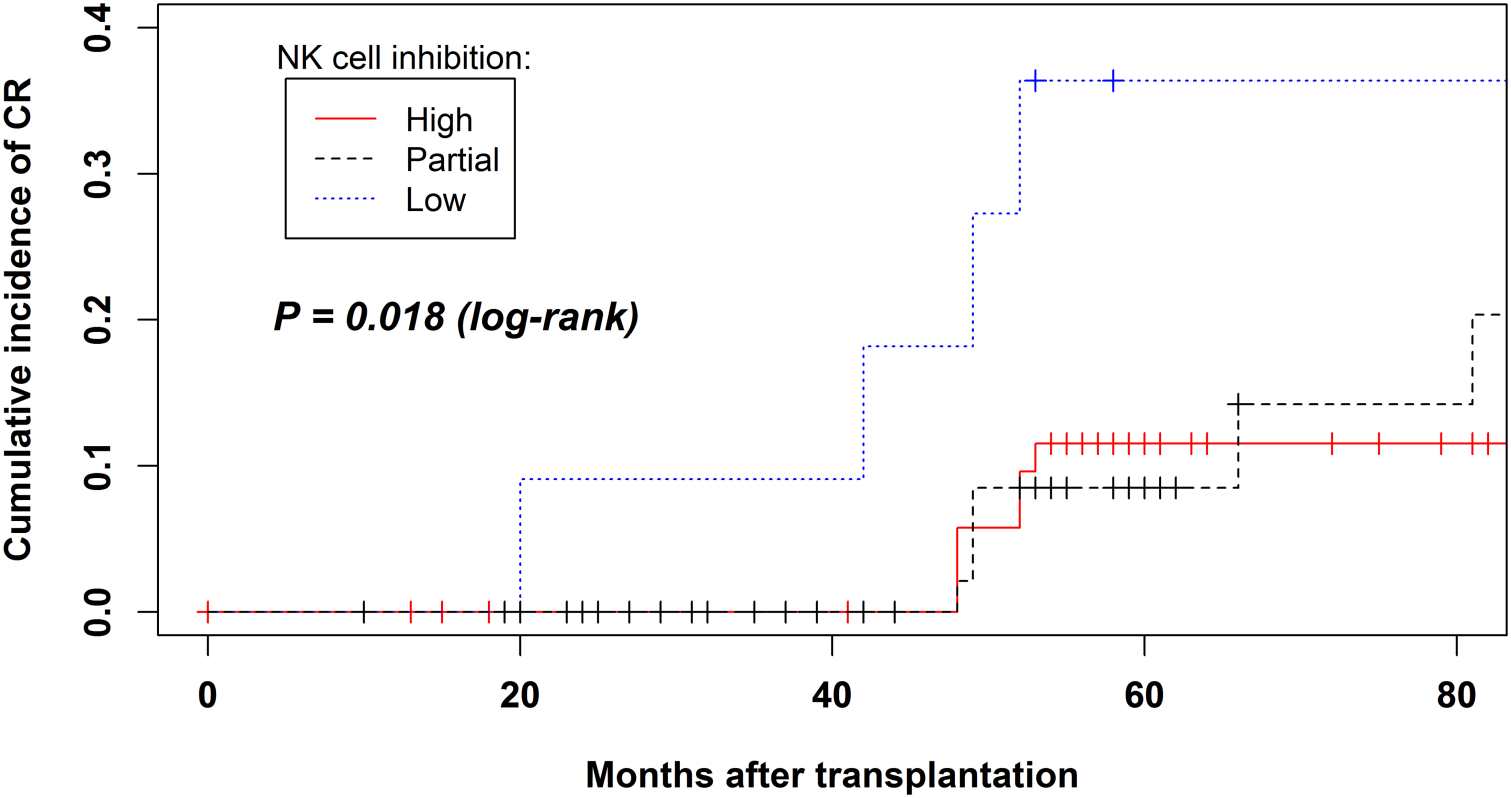
Kaplan-Meier cumulative incidence of chronic rejection in the three groups of patients stratified according to the presence or absence of one or both of the following combinations of recipient (r) KIRs and donor (d) HLA ligands: rKIR2DL1/dHLA-C2 and rKIR3DL1/dHLA-Bw4. Patients with both combinations = high NK cell inhibition; patients with one combination = partial NK cell inhibition; patients lacking both combinations = low NK cell inhibition.

The Kaplan-Meier curves clearly show that the cumulative incidence of chronic rejection is significantly higher in the group of patients completely lacking these combinations (low NK cell inhibition) compared to patients of the other two groups (log rank = 0.018; Fig 1).

**Fig 2:**
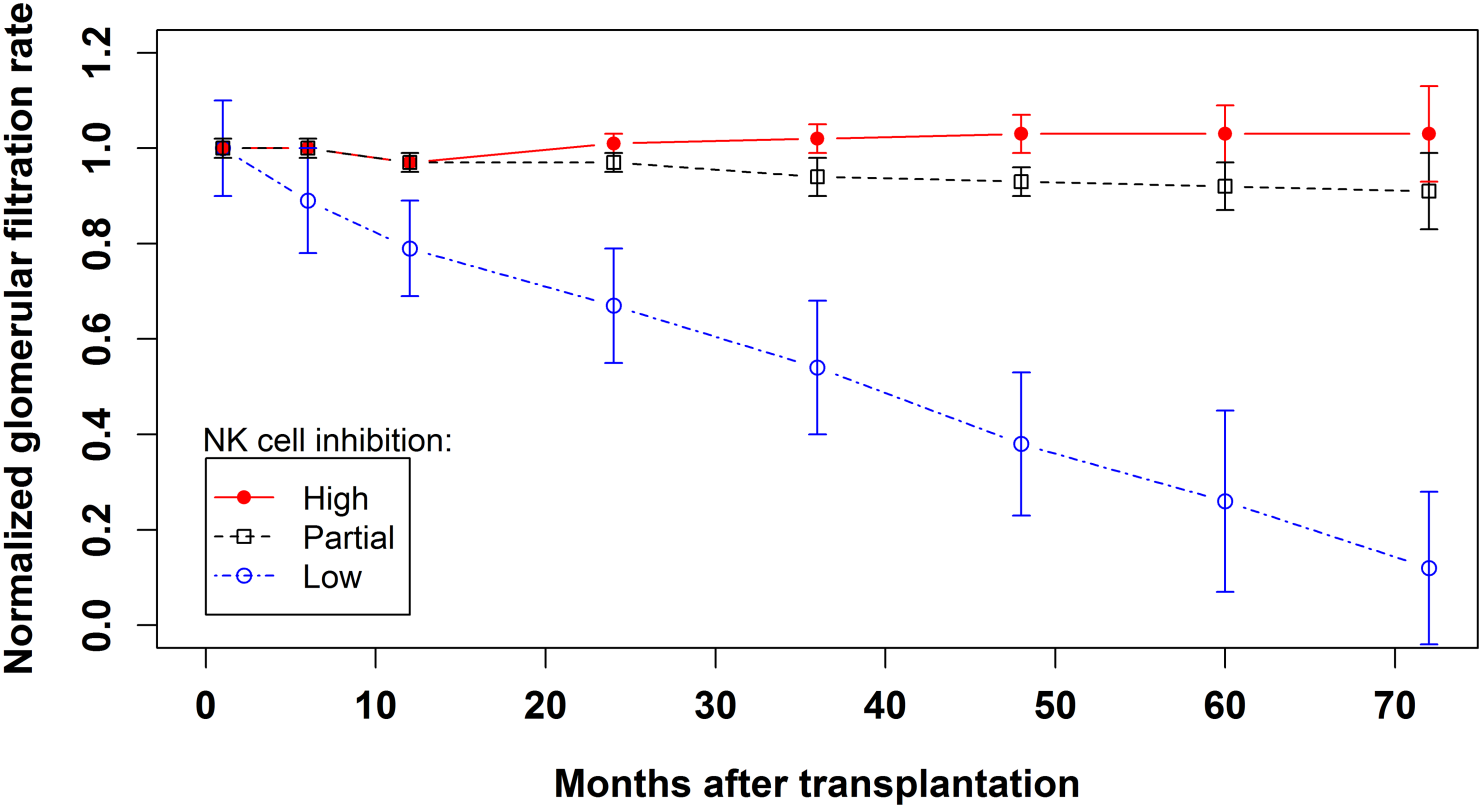
Glomerular filtration rate measured at 1, 6, 12, 24, 36, 48, 60 and 72 months after transplantation normalized to baseline levels measured at 1 month after transplantation. The error bar at each point represents the 95% Confidence Interval. The group of patients with low NK cell inhibition (completely lacking the two functional units rKIR2DL1/dHLA-C2 and rKIR3DL1/dHLA-Bw4) showed a statistically significant difference in comparison to the groups of patients with either partial or high NK cell inhibition; P = 3.5·10^−14^ obtained by calculating the area under the curve.

Modification of Diet in Renal Disease (MDRD)-estimated GFR confirmed the influence on graft function of the absence of rKIR2DL1/dHLA-C2 and rKIR3DL1/dHLA-Bw4 (low NK cell inhibition). In fact, patients with this low NK cell inhibition profile displayed an overall decline in GFR that was significantly stronger than in patients with high or partial NK cell inhibition profiles (P = 3.5 x 10^−14^). More specifically, at 12 months after transplantation, a progressively stronger decline in MDRD-estimated GFR was observed in the group of patients with low NK cell inhibition. The statistically significant difference observed at 24 months (10.1 ml/min per 1.73 m2, 95% CI 6.4–13.8, P = 2.8 x 10^−7^) continued to rise at 36 (18.8 ml/min per 1.73 m2, 95% CI 13.6–23.9, P = 4.3 x 10^−11^) and 72 months (49.0 ml/min per 1.73 m2, 95% CI 36.4–61.7, P = 3.7 x 10^−9^) after transplantation (Fig 2).

Also serum creatinine levels correlated with the presence or absence of rKIR2DL1/dHLA-C2 and rKIR3DL1/dHLA-Bw4. Mean serum creatinine levels in patients with low NK cell inhibition were significantly higher than those of patients with high or partial NK cell inhibition at 24 months and continued to increase at 36 and 72 months (scr = 1.76 vs 1.32 mg/dL, P = 0.02; scr = 2.40 vs 1.31 mg/dL, P = 6.5 x 10^−4^; scr = 6.20 vs 1.28 mg/dL, P = 3.4 x 10^−10^), respectively (Table 6).

**Table 6:** Serum creatinine levels after transplantation in three groups of patients stratified according to the presence or absence of combinations of recipient (r) KIRs and donor (d) HLA ligands: rKIR2DL1/dHLA-C2 and rKIR3DL1/dHLA-Bw4.

Serum Creatinine mg/dL (mean ± standard deviation)								
**rKIR2DL1/dHLA-C2** **rKIR3DL1/dHLA-Bw4:**								
High NK cell inhibition (**+/+**)	1.40 ± 0.48	1.40 ± 0.42	1.45 ± 0.54	1.35 ± 0.51	1.30 ± 0.88	1.32 ± 0.39	1.29 ± 0.67	1.30 ± 0.70
Partial NK cell inhibition (**+/- or -/+**)	1.27 ± 0.58	1.29 ± 0.40	1.20 ± 0.53	1.28 ± 0.47	1.32 ± 0.46	1.25 ± 0.68	1.22 ± 0.53	1.19 ± 0.78
Low NK cell inhibition (**-/-**)	1.12 ± 0.42	1.28 ± 0.67	1.55 ± 0.58	1.76 ± 0.98	2.40 ± 1.14	2.72 ± 1.35	4.65 ± 1.68	6.20 ± 1.57
**P value**	0.15	0.58	0.21	**0.02**	**6.5·10**^**−4**^	**8.6·10**^**−7**^	**3.8·10**^**−9**^	**3.4·10**^**−10**^
**Time (months)**	1	6	12	24	36	48	60	72

P values were calculated by comparing serum creatinine levels of patients without rKIR2DL1/dHLA-C2 and rKIR3DL1/dHLA-Bw4 (low NK cell inhibition) vs patients with the other two combinations (partial or high NK cell inhibition).

Analysis of combinations of rKIR2DL2/L3-dHLA-C1 did not yield any statistically significant differences between patients with chronic rejection and patients with stable graft function (S2 Table). These results were confirmed by Kaplan-Meier curves (S1 Fig).

## Discussion

Incompatibility for the *HLA-A*, *-B* and–*DR* class I and II loci remains the major obstacle to the successful outcome of kidney transplantation. Alongside the humoral immunological response mechanisms responsible for chronic antibody-mediated rejection, HLA mismatches between donors and recipients promotes the activation of CD4+ and CD8+ T cell-mediated effector pathways that lead to a cascade of events causing graft damage and the clinical symptoms of chronic rejection [4].

An interesting finding in our study was the high frequency observed for HLA-*A24 (P = 5.0 x 10^−6^) and HLA-*B14 (P = 0.002) in donors of patients with chronic rejection compared to donors of patients with stable graft function. To our knowledge, there are no reports in the literature describing association of these two alleles with rejection. However, it has been reported that HLA-*A24 is capable of increasing response of cytotoxic CD8+ T cells, thereby protecting individuals with this allele against H1N1 influenza A viral infection [41] and Epstein-Barr virus [42]. Moreover, HLA-*A24 has been used within a pool of antigens for the study of human T cell response in autoimmune diseases [43]. This once more confirms that certain HLA antigens of the donor may increase host immune response against the graft and thus promote the onset of chronic rejection.

In the literature, there is an abundance of information describing the influence of NK cell alloreactivity on the outcome of kidney transplantation. Vampa et al. performed a study in vitro that demonstrated an increment of NK cell cytotoxicity after transplantation in patients with an activating KIR gene specific for HLA class I ligands of the graft [11]. Nowak et al. found that the KIR2DS5 activating receptor conferred protection against onset of acute rejection, particularly in patients lacking the deletion variant of the *KIR2DS4* activating gene [8, 44]. This protective effect was not confirmed by Kunert et al. who instead found that inhibitory rather than activating KIR genes were capable of reducing the risk of acute graft rejection [45].

In our study, we did not find a protective effect for the *KIR2DS5* activating KIR gene against chronic rejection, analyzed both alone or in combination with the full length *KIR2DS4* gene. However, this result may possibly be explained by diversity in the immunogenetic mechanisms underlying acute and chronic rejection [46].

Overall, our data suggest a role for inhibitory KIR genes and their ligands in potentiating the risk of chronic rejection and reducing long term graft survival. First of all, we observed a highly significant frequency for C1 homozygous donors in the group of patients with chronic rejection (Table 5). Binding and functional studies show that the strengths of KIR/HLA-C inhibitory interactions vary. In particular, KIR2DL1 interaction with HLA-C2 is considered to be the combination with the strongest inhibitory effect whereas KIR2DL2/L3 interactions with HLA-C1 confer weaker inhibitory effects [47]. Therefore, allografts that only express HLA antigens of the C1 group may directly increase cytotoxicity of NK cells or activate other effector pathways.

Kunert et al. describe better outcomes in patients with grafts from donors homozygous for HLA-C group C2 alleles (P = 0.052). This is probably because interaction between donor-derived NK cells and recipient dendritic cells occurs with more feeble activation of indirect alloreactivity in HLA-C2 than in HLA-C1 positive recipients [45, 48].

**Fig 3:**
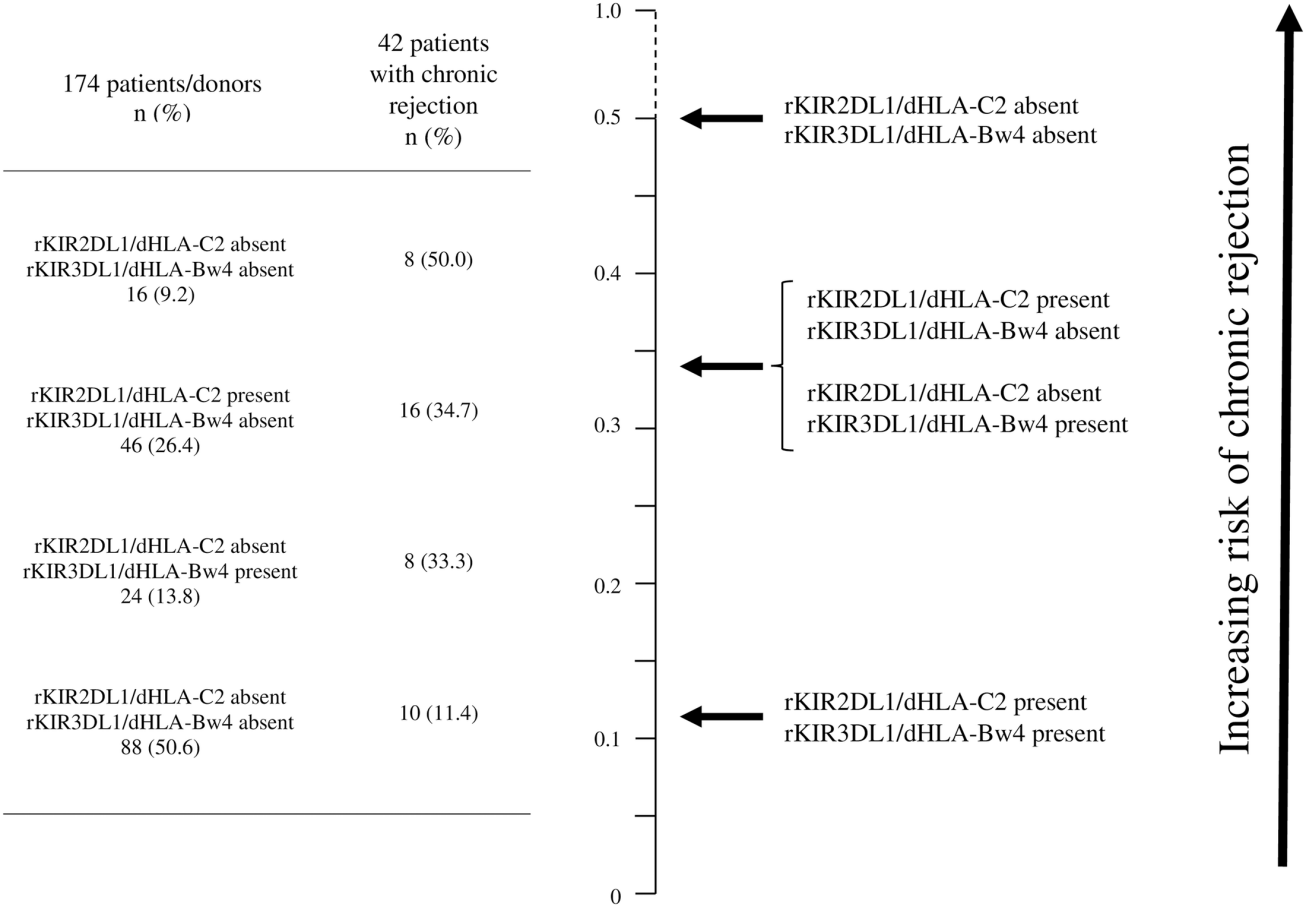
Risk of chronic rejection according to the presence or absence of specific recipient (r) KIR and donor (d) HLA ligand combinations.

Another relevant finding in our study was that patients with chronic rejection had significantly reduced frequencies of rKIR2DL1/dHLA-C2 and rKIR3DL1/dHLA-Bw4 (Table 5). This immunogenetic profile may determine an increase in the number of uneducated NK cells, which besides being less potentiated are less sensitive to inhibition by self HLA (Fig 3).

The Kaplan-Meier curves clearly show the increased risk for chronic rejection in patients with low NK cell inhibition (lacking the rKIR2DL1/dHLA-C2 and rKIR3DL1/dHLA-Bw4 functional units) compared to those with either partial or high NK cell inhibition (Fig 1).

Immunosuppressive drug regimens are at least partially capable of containing the negative effects of this immunogenetic profile, particularly in the early post-transplantation phase. Nonetheless, at 12 months after transplantation, GFR in kidney graft recipients completely lacking the KIR2DL1/ dHLA-C2 and KIR3DL1/dHLA-Bw4 functional units was generally lower than in patients with one or both of these combinations. This difference progressively increased, reaching statistical significance at 24 months after transplantation (Fig 2).

The absence of both these functional units was not a frequent finding in our cohort of patients (16/174, 9.2%), nor in our group of 221 controls (22/221, 10.0%). Indeed, the presence of NK cells deprived of functional receptor-ligand units critical to their activity may create an imbalance between activating and inhibitory signals and consequential defects in NK cell-mediated cytolysis.

In agreement with the current literature, it is possible that such subpopulations of NK cells promote the development of chronic rejection by accelerating maturation and migration of graft-infiltrating host dendritic cells (DC). Priming of the adaptive immune system via NK-DC immune synapse leads to T-helper type-1 (Th1)-dominated immune response mechanisms [48, 49]. Moreover, NK-DC interaction is facilitated by the high levels of interleukin-15 (IL-15) expressed by the kidney in the early post-transplantation stage [50].

In our study, the selection of recipient and donor pairs fully matched for the DR locus was aimed at minimizing the effects of humoral and cell-mediated adaptive immunity in order to better evaluate the impact of NK cell alloreactivity on the development of chronic rejection. Ten percent of our patients had low levels of NK cell inhibition (completely lacking the two functional combinations rKIR2DL1/dHLA-C2 and rKIR3DL1/dHLA-Bw4) and a higher risk of chronic rejection. Such patients could benefit from immunosuppressive therapy based on alternative drugs capable of reducing NK-cell cytotoxicity, such as rapamycin and mycophenolate [51]. However, further study will be required to evaluate the impact of these findings on the large majority of patients transplanted from HLA-DR mismatched donors.

Overall, our data add two more pieces to the complex puzzle of cell-mediated chronic rejection and loss of graft function, which may contribute to current practices of risk assessment and help optimize the results of kidney transplantation.

## Supporting information

S1 Fig Kaplan-Meier curves obtained by dividing the patients into 3 groups according to the presence or absence of one or both of the combinations of recipient (r) KIRs and donor (d) HLA ligands rKIR2DL2/L3-dHLA-C1, investigated for association with chronic rejection. (TIFF)

S1 Table HLA Class I allele frequencies in cadaveric kidney donors, patients and healthy controls. (DOCX)

S2 Table Presence or absence of one or both of the combinations of recipient (r) KIRs and donor (d) HLA ligands rKIR2DL2/L3-dHLA-C1, investigated for association with chronic rejection. CR = chronic rejection, SGF = stable graft function.
(DOCX)
