# Supplementary figures and images for: KIR and their HLA Class I ligands: Two more pieces towards completing the puzzle of chronic rejection and graft loss in kidney transplantation
Source: PLoS One. 2017 Jul 7;12(7):e0180831. doi: 10.1371/journal.pone.0180831 (PMC5501603; doi:10.1371/journal.pone.0180831)

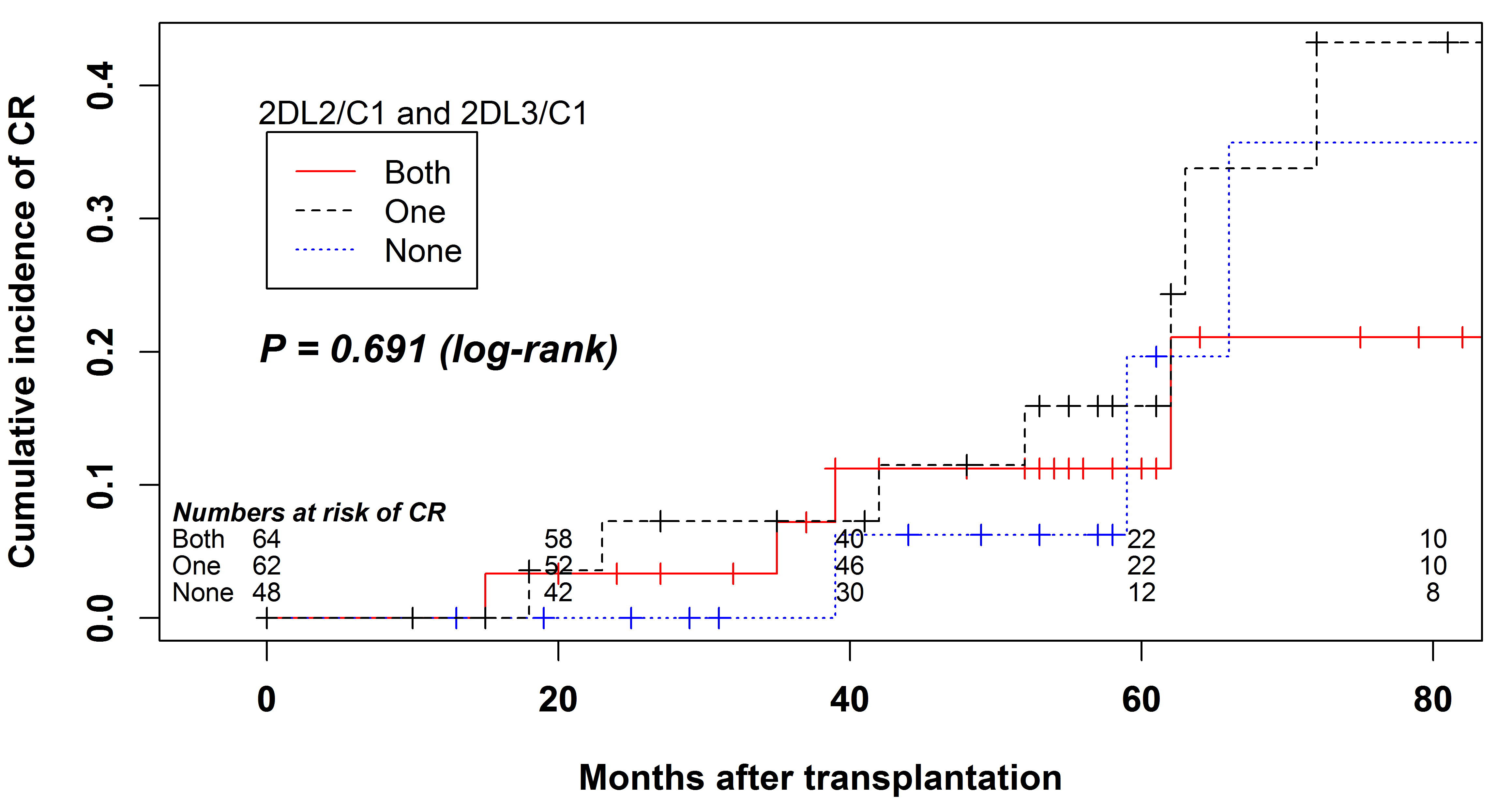

Supplement: S1 Fig — (TIFF) [file pone.0180831.s001.tiff]
