# Supplementary material for: KIR and their HLA Class I ligands: Two more pieces towards completing the puzzle of chronic rejection and graft loss in kidney transplantation
Source: PLoS One. 2017 Jul 7;12(7):e0180831. doi: 10.1371/journal.pone.0180831 (PMC5501603; doi:10.1371/journal.pone.0180831)
Supplement: S1 Table — (DOCX) [file pone.0180831.s002.docx]

|  | **HLA**  **(2388 controls; 4776 alleles)** | **HLA**  **(174 patients; 348 alleles)** | **Controls**  **vs**  **Patients** | **HLA**  **(174 donors;**  **348 alleles)** |  | **Donors**  **vs**  **Patients** |
| --- | --- | --- | --- | --- | --- | --- |
|  | **n (%)** | **n (%)** | **P value** | **n (%)** |  | **P value** |
| **HLA-A allele frequencies** | | | | | | |
| -A*01 | 363 (7.6) | 26 (7.5) | 1 | 24 (6.9) |  | 0.883 |
| -A*02 | 1256 (26.3) | 100 (28.7) | 0.315 | 104 (29.9) |  | 0.803 |
| -A*03 | 282 (5.9) | 21 (6.0) | 0.906 | 20 (5.7) |  | 1 |
| -A*11 | 397 (8.3) | 29 (8.3) | 1 | 28 (8.0) |  | 1 |
| -A*23 | 82 (1.7) | 1 (0.3) | 0.044 | 0 (0) |  | 1 |
| -A*24 | 444 (9.3) | 34 (9.8) | 0.774 | 34 (9.8) |  | 1 |
| -A*26 | 134 (2.8) | 13 (3.7) | 0.316 | 16 (4.6) |  | 0.705 |
| -A*29 | 93 (1.9) | 3 (0.9) | 0.215 | 2 (0.6) |  | 1 |
| -A*30 | 851 (17.8) | 55 (15.8) | 0.382 | 52 (14.9) |  | 0.834 |
| -A*31 | 66 (1.4) | 3 (0.9) | 0.627 | 4 (1.1) |  | 1 |
| -A*32 | 419 (8.8) | 34 (9.8) | 0.495 | 34 (9.8) |  | 1 |
| -A*33 | 193 (4.0) | 20 (5.7) | 0.126 | 20 (5.7) |  | 1 |
| -A*68 | 96 (2.0) | 5 (1.4) | 0.687 | 4 (1.1) |  | 1 |
| -A*69 | 51 (1.1) | 0 (0) | 0.048 | 0 (0) |  | 1 |
| -A- | 49 (1.0) | 4 (1.1) | 0.781 | 6 (1.7) |  | 0.752 |
| **HLA-C allele frequencies** | | | | | | |
| -Cw*01 | 161 (3.4) | 15 (4.3) | 0.358 | 16 (4.6) |  | 1 |
| -Cw*02 | 299 (6.3) | 22 (6.3) | 0.909 | 20 (5.7) |  | 0.874 |
| -Cw*03 | 266 (5.6) | 20 (5.7) | 0.904 | 20 (5.7) |  | 1 |
| -Cw*04 | 633 (13.3) | 53 (15.2) | 0.290 | 56 (16.1) |  | 0.835 |
| -Cw*05 | 838 (17.5) | 68 (19.5) | 0.344 | 70 (20.1) |  | 0.924 |
| -Cw*06 | 320 (6.7) | 28 (8.0) | 0.321 | 28 (8.0) |  | 1 |
| -Cw*07 | 1095 (22.9) | 81 (23.3) | 0.895 | 80 (23.0) |  | 1 |
| -Cw*08 | 360 (7.5) | 26 (7.5) | 1 | 24 (6.9) |  | 0.883 |
| -Cw*12 | 364 (7.6) | 23 (6.6) | 0.599 | 24 (6.9) |  | 1 |
| -Cw*14 | 66 (1.4) | 0 (0) | 0.022 | 0 (0) |  | 1 |
| -Cw*15 | 221 (4.6) | 12 (3.4) | 0.353 | 10 (2.9) |  | 0.829 |
| -Cw*16 | 102 (2.1) | 0 (0) | 0.001 | 0 (0) |  | 1 |
| -Cw*17 | 51 (1.1) | 0 (0) | 0.048 | 0 (0) |  | 1 |
| **HLA-B allele frequencies** | | | | | | |
| -B*07 | 132 (2.8) | 4 (1.1) | 0.082 | 4 (1.1) |  | 1 |
| -B*08 | 92 (1.9) | 7 (2.0) | 0.840 | 6 (1.7) |  | 1 |
| -B*13 | 109 (2.3) | 9 (2.6) | 0.710 | 8 (2.3) |  | 1 |
| -B*14 | 369 (7.7) | 27 (7.8) | 1 | 28 (8.0) |  | 1 |
| -B*15 | 88 (1.8) | 0 (0) | 0.004 | 0 (0) |  | 1 |
| -B*18 | 1066 (22.3) | 82 (23.6) | 0.594 | 82 (23.6) |  | 1 |
| -B*27 | 113 (2.4) | 6 (1.7) | 0.580 | 6 (1.7) |  | 1 |
| -B*35 | 661 (13.8) | 58 (16.7) | 0.150 | 60 (17.2) |  | 0.920 |
| -B*37 | 65 (1.4) | 7 (2.0) | 0.338 | 8 (2.3) |  | 1 |
| -B*38 | 80 (1.7) | 10 (2.9) | 0.133 | 12 (3.4) |  | 0.829 |
| -B*39 | 103 (2.2) | 13 (3.7) | 0.062 | 14 (4.0) |  | 1 |
| -B*40 | 77 (1.6) | 0 (0) | 0.010 | 0 (0) |  | 1 |
| -B*41 | 53 (1.1) | 3 (0.9) | 1 | 2 (0.6) |  | 1 |
| -B*44 | 221 (4.6) | 12 (3.4) | 0.353 | 12 (3.4) |  | 1 |
| -B*45 | 54 (1.1) | 4 (1.1) | 1 | 2 (0.6) |  | 0.686 |
| -B*49 | 217 (4.5) | 16 (4.6) | 0.894 | 14 (4.0) |  | 0.852 |
| -B*50 | 59 (1.2) | 8 (2.3) | 0.134 | 8 (2.3) |  | 1 |
| -B*51 | 343 (7.2) | 18 (5.2) | 0.192 | 18 (5.2) |  | 1 |
| -B*52 | 83 (1.7) | 4 (1.1) | 0.523 | 6 (1.7) |  | 0.752 |
| -B*55 | 178 (3.7) | 10 (2.9) | 0.553 | 10 (2.9) |  | 1 |
| -B*57 | 43 (0.9) | 1 (0.3) | 0.365 | 0 (0) |  | 1 |
| -B*58 | 436 (9.1) | 30 (8.6) | 0.847 | 28 (7.8) |  | 0.891 |
| -B- | 134 (2.8) | 19 (5.5) | 0.009 | 20 (5.7) |  | 1 |

SGF = stable graft function, CR = chronic rejection
