# Supplementary material for: KIR and their HLA Class I ligands: Two more pieces towards completing the puzzle of chronic rejection and graft loss in kidney transplantation
Source: PLoS One. 2017 Jul 7;12(7):e0180831. doi: 10.1371/journal.pone.0180831 (PMC5501603; doi:10.1371/journal.pone.0180831)
Supplement: S2 Table — CR = chronic rejection, SGF = stable graft function. (DOCX) [file pone.0180831.s003.docx]

| **2DL2/C1**  **and 2DL3/C1:**  **presence or**  **absence** | **174 patients**  **n (%)** | **42 patients**  **with CR**  **n (%)** | **132 patients**  **with SGF**  **n (%)** | **OR (95% CI)** | **P value** |
| --- | --- | --- | --- | --- | --- |
| Both present | 64 (36.8) | 10 (23.8) | 54 (40.9) | 0.45 (0.18 – 1.04) | 0.065 |
| One present | 62 (35.6) | 20 (47.6) | 42 (31.8) | 1.94 (0.90 – 4.19) | 0.068 |
| None present | 48 (27.6) | 12 (28.6) | 36 (27.3) | 1.07 (0.45 – 2.43) | 0.846 |
